# The fundamental challenge of a future theory of consciousness

**DOI:** 10.3389/fpsyg.2022.1029105

**Published:** 2023-01-12

**Authors:** Zenan Ruan

**Affiliations:** ^1^Zhejiang University—University of Luxembourg Joint Laboratory on AIs, Robotics and Reasoning (ZLAIRE), School of Philosophy, Zhejiang University, Hangzhou, China; ^2^Department of Philosophy of Science and Technology, School of Philosophy, Zhejiang University, Hangzhou, China

**Keywords:** consciousness, models of consciousness, theories of consciousness, IIT, GNWT

## Introduction

In the wide field of cognitive science, there are many works on consciousness. These studies can be roughly divided into two categories, i.e., the philosophical (especially the philosophy of mind) and the scientific (especially cognitive neuroscience). Neuroscientists have been searching for “neural correlates of consciousness” (NCCs, cf. Koch, 2012; Tononi and Koch, 2015), an approach that prioritizes the study of the correlation between specific brain activity and aspects of consciousness, while a philosophical approach always focuses on identifying explanatory links between neural mechanisms and consciousness.

The philosophical approach to consciousness was at one point difficult due to the “hard problem” argued by Chalmers (1995), that is, there seems to be an unbridgeable gap between the first-person perspective and the third-person perspective. Nonetheless, the current empirical Theories of Consciousness (ToCs) lay in an unoptimistic situation, where they “talk” past each other (Seth and Bayne, 2022; Yaron et al., 2022). As the number of theories keeps increasing (Signorelli et al., 2021), certain projects like adversarial collaboration (Melloni et al., 2021) are proposed and valued. A theory [for a preliminary case, see Safron (2020)] is expected to eventually surpass such situations in the future (Koch, 2018), yet there remain challenges to be met.

## Criteria for the competition of various theories

Since Crick and Edelman, some neuroscientists have tended to support setting aside the “hard problem” and starting with the easy ones. Ideally, testing, contrasting, and iterating those ToCs might ultimately lead to a deeper understanding of the mystery of consciousness. Nevertheless, all theories need not be directly adopted to empirical tests without effective constraints.

As a beginning, Doerig et al. (2021) proposed four challenges that constrain current theories, the so-called “hard criteria,” which are “hard” on their emphasis on rigor and articulation and different from some evaluations that are vague and difficult to compare effectively. Overall, their initiations and contributions seeking empirical standards of ToCs are inspiring, but there are certain criticisms of their specific proposals, especially those that treat consciousness only as a function (Del Pin et al., 2021). As for discussions on conscious experience (Edelman and Tononi, 2001) in terms of its states and contents are concerned, they appear to be overlapping—an experience (as identified by its state) is always created along with at least one of the specific aspects of contents (cf. Damasio, 2021). Thus, functionalists tend to take much count of the contents of consciousness (Fleming, 2021) that are typically revealed in experiments through verbal or behavioral reports—several kinds of measurements. Even in the no-report paradigm (Tsuchiya et al., 2015; Koch et al., 2016), we cannot say certainly that we captured the experiential markers of the subjects, which can exclude the cognitive processes that are the consequences of consciousness (Northoff and Huang, 2017).

Recently, Seth and Bayne (2022) explicitly surveyed four typical ToCs based on their theoretical origination, the basis for prediction, and the specific problems they target. From these analyses, an avenue from the various theories toward a comprehensive and satisfactory theory should be to supply more comparable explanatory objects and discussion bases (such as the preference for the phenomenological or functional characteristics of consciousness and the global or local states of consciousness). Furthermore, they proposed three conditions for effective consideration of any feasible future theory referring to explicitness, comprehensiveness, and measurement, respectively.

Constraints for the study of consciousness are a form of meta-theoretical study that certainly makes sense, and in principle, we need to address them all. Moreover, such criteria may be as great in number as the diversity of theories of consciousness itself. We should consider the fundamental criterion underlying these challenges and why it matters. How, or at least to what extent, might the empirical rivalries between the various ToCs ultimately iterate to a “true” theory?

## Criterion necessary and sufficient for the models of consciousness

Specific models in ToCs describe experiences from a particular point of view, and some of them are less rigorous, especially when they usually originate only from a single empirical finding and reflection. For an early example, inspired by the regular discharge in certain conscious activities, Crick and Koch concluded that the cortical mechanism responsible for gamma oscillations could be the NCC (Koch, 2019, p. 98–100). In addition, the NMDA theory was proposed because the NMDA synapses were found to work along with conscious experience (Flohr, 1992; Doerig et al., 2021).

In most cases, these synapses are all acceptable. Experiments within a theory can still be kept going as long as other diverse theories are set aside. After all, thought experiments of Chalmers could not have “jumped out” as a threat to their independent empirical experiment. Given the comprehensive considerations, N–S (necessary and sufficient), a fundamental criterion for a qualified model of consciousness that is necessary and sufficient. If the description of a ToC meets the N–S criterion, then its model would be entirely identical to consciousness itself. In the empirical test, its predictions of a subject would not be inconsistent with what it indeed is.

**Figure 1:**
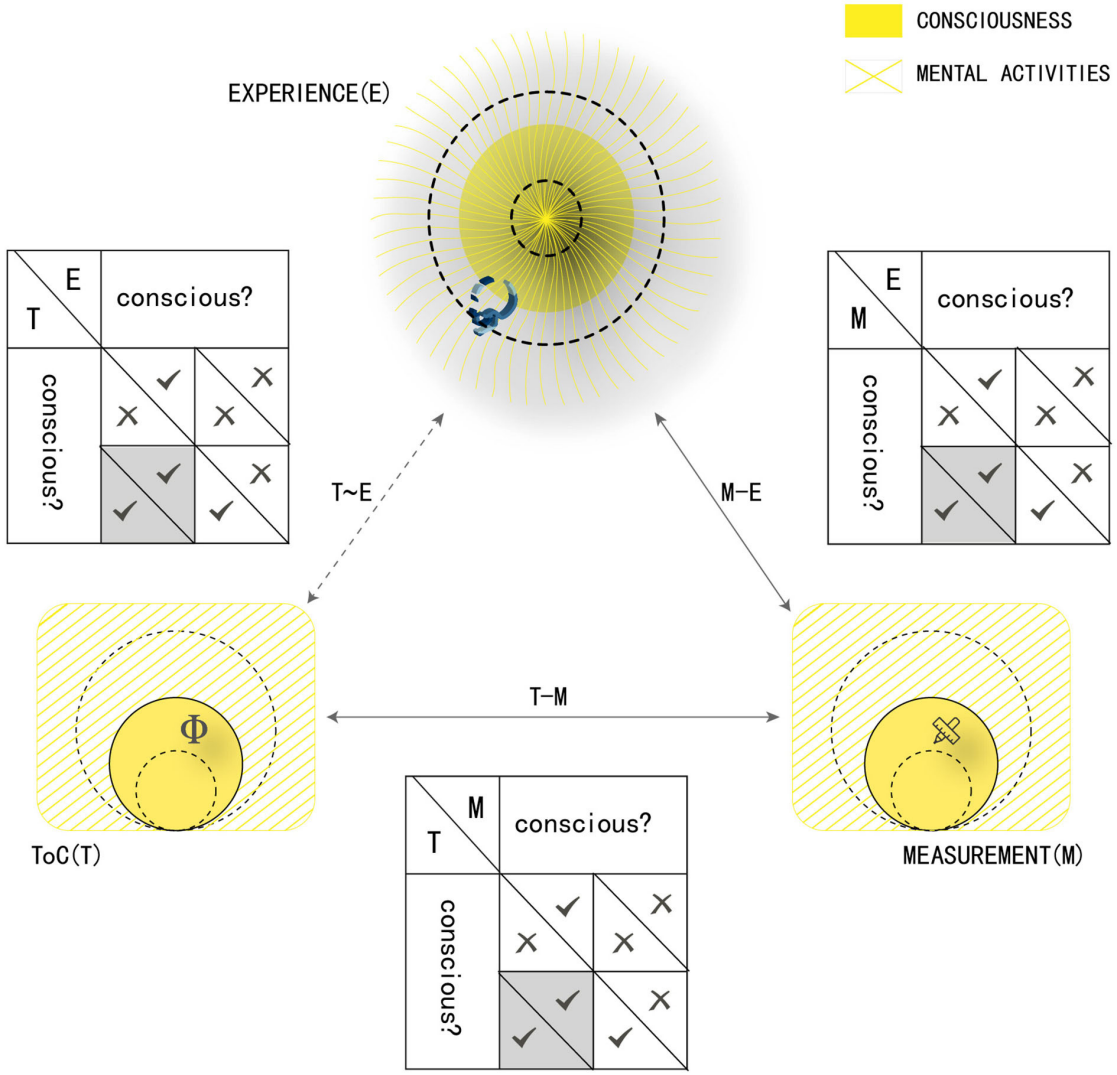
The N–S criterion of ToCs. (1) The general: A “proper” ToC specifies a model identical to consciousness, whose “necessity” is to get a fairly “wide” scope covering all the mental activities and whose “sufficiency” is to contain a “narrow” enough scope of those mental activities (see **bottom left**, ToC, denoted as T). Both the “wide” and the “narrow” are shown by dashed circles, while the filled portion within the solid circles is the necessary and sufficient region. (2) The specific: Similarly, the experience itself (**top**, denoted as E) and the measurement (**bottom right**, denoted as M) also get in the proper scope of where the consciousness is located throughout the mental activity. E is considered proper a priori and the identity between T and E (T~E, ~ for a transcendental relation) is the goal of the traditional philosophical approaches. Theoretically, only one (gray-filled) of the four underlying relations between them can meet the N–S criterion. For empirical tests, three levels of the N–S criterion (T–M, M–E, and then T–M–E) are a must and each of level would constrain the corresponding procedure of the ToC tests successively.

However, this intuitive insight would not yet bring us much in practice. In a sense, it is an empirical version of the “hard problem.” Furthermore, we must note that there would be a triple identity of the expected equation “theoretical model = consciousness” from an empirical perspective, corresponding the model to the hypothetical consciousness (T), the data of measurements (M), and the experience itself of the subject (E), respectively (see Figure 1). The most straightforward solution is the traditional philosophical approach from T to E (T~E), which may have been mired in the “hard problem.” The empirical approach to consciousness consequently has to go through the “proxy” of measurement (T–M–E) to the extent that such a complex situation emerges.

## Three levels of the N–S criterion

Considering the practical details, the N–S criterion needs to delineate three progressive levels due to the triple identity of consciousness in empirical tests.

1. The candidate theories

A theory of consciousness is completely self-consistent inherently and eligible for experiments. Because of the inappropriate thinking of the proponents or the limited resource or data, the proposals of some theories may have obvious or obscure logical weaknesses or may be contrary to the recognized laws of nature. In such situations, the theories would be first eliminated or need to be improved further. Then, rather than being mysterious, their models must be experimentally measurable. They need to be also precise enough in their description for they must be able to address most aspects (Doerig et al., 2021) of the discussion on consciousness. Through such self-examination, a theory would meet the first level of the N–S, referring to the relation T–M (see Figure 1, bottom). Thus, they qualify to be the candidates and can undergo valid empirical tests with their own assumptions independently.

The first level is usually met relatively easily. Theories that tentatively meet the first level of the N–S criterion may retain their place in the study of consciousness. For example, the Integrated Information Theory (IIT), once with the so called “coarse-graining problem” (Mørch, 2018), has been initially applied to measure the consciousness of patients clinically.

2. Intra-paradigm tests

When a theory meeting the first level has undergone adequate experiments independently, it could be ready for the second level. Here, those candidate theories with the same assumptions have to be tested together as competitors for their experiments with the same paradigms. The relation M–E (Figure 1, right) is referred to by this level in the empirical tests.

There is clearly no generally accepted recognition of what consciousness exactly is. Thus, diverse theories could prefer to have their own choices. For example, the Global Workspace Theory (GWT, cf. Mashour et al., 2020) has fixed it to “conscious access”, while IIT has fixed it to phenomenal consciousness. Whatever their preferences, they must be rigorously held accountable for their choices and make corrections when the empirical findings deviate from the fixed goals. In this case, the paradigm of IIT should try to avoid the interference of typical cognitive functions such as language and working memory. Instead, IIT and the Recurrent Processing Theory (RPT, cf. Lamme, 2006) may be considered a valid adversarial collaboration under the specific paradigm, as both of them are causal structure theories and have asserted no necessity for frontal regions.

Briefly, the constraint imposed by the N–S criterion at this level can be dedicated to making those similar theories compete effectively together. Given a fixed description of consciousness, the predictions of an ideal theory under the paradigm should be kept consistent with all the experimental results.

3. Tests between paradigms

Constraints at this level require the consciousness fixed in its experiments by a theory is indeed the subjects' experience. As illustrated, the total identity T–M–E (Figure 1, top) is referred to by this level. In a sense, it requires transforming the “hard problem” to be a series of hard but promising work (Baars, 2021).

A certain set of theories may remain logical and tested well within their own paradigm, without considering the metaphysics of consciousness or their meta-level. Therefore, these ToCs with diverse paradigms would completely be eligible to start this one and compete with each other after they have met the constraints of the first two levels of the N–S criterion. This is where the rivalry between IIT and GWT could finally come in if they would have both succeeded in their own paradigm. The competitions are indeed some of the preferences to model the consciousness in each theory. In other words, which choice is indeed the most precise—or closer to consciousness itself?

## Conclusion

Chalmers' “hard problem” does point out a central problem in the study of consciousness, while his thought experiments are always ignored in empirical experiments. Thus, the N–S criterion would be its concrete, empirically enhanced version and highlights especially the assumptions and paradigms in the experiments. In fact, the criterion and all its specific levels are concluded from various arguments on the constraints of the study of consciousness.

The final level partly reflects the requirement of the paradigm shift toward first-person science (Ascoli, 2013; Ellia et al., 2021; Pinto and Stein, 2021). Studying subjective phenomena can be possible, where we have internal measurements like “first-hand experience” (Kleiner, 2020), which allows scientists to first be used to introspection or meditation. Perhaps, some inspiration may come from the “interhemispheric communication” (Watanabe, 2022) between the referee and the measured variables.

The science of consciousness may transcend the current one, but it is also fine now. Our opinion is that the tests of the various theories of consciousness should not be a one-step kind test. As the level of the fundamental criteria increases in tests, a further theory would be more accurate, at least in the sense of probability and chance. The opinion aims at an initiative for further discussion of constraints in the empirical tests of contemporary ToCs.

## Author contributions

ZR conceived the opinion and wrote the entirety of the manuscript.
